# The Viral Oncoprotein LMP1 Exploits TRADD for Signaling by Masking Its Apoptotic Activity

**DOI:** 10.1371/journal.pbio.0060008

**Published:** 2008-01-15

**Authors:** Frank Schneider, Julia Neugebauer, Janine Griese, Nicola Liefold, Helmut Kutz, Cinthia Briseño, Arnd Kieser

**Affiliations:** Department of Gene Vectors, GSF–National Research Center for Environment and Health, Munich, Germany; University of Wisconsin-Madison, United States of America

## Abstract

The tumor necrosis factor (TNF)-receptor 1–associated death domain protein (TRADD) mediates induction of apoptosis as well as activation of NF-κB by cellular TNF-receptor 1 (TNFR1). TRADD is also recruited by the latent membrane protein 1 (LMP1) oncoprotein of Epstein-Barr virus, but its role in LMP1 signaling has remained enigmatic. In human B lymphocytes, we have generated, to our knowledge, the first genetic knockout of *TRADD* to investigate TRADD's role in LMP1 signal transduction. Our data from TRADD-deficient cells demonstrate that TRADD is a critical signaling mediator of LMP1 that is required for LMP1 to recruit and activate I-κB kinase β (IKKβ). However, in contrast to TNFR1, LMP1-induced TRADD signaling does not induce apoptosis. Searching for the molecular basis for this observation, we characterized the 16 C-terminal amino acids of LMP1 as an autonomous and unique virus-derived TRADD-binding domain. Replacing the death domain of TNFR1 by LMP1′s TRADD-binding domain converts TNFR1 into a nonapoptotic receptor that activates NF-κB through a TRAF6-dependent pathway, like LMP1 but unlike wild-type TNFR1. Thus, the unique interaction of LMP1 with TRADD encodes the transforming phenotype of viral TRADD signaling and masks TRADD's pro-apoptotic function.

## Author Summary

For viral infection to succeed, viral proteins must interact with the cellular signaling machinery of its target cell. An oncoprotein encoded by the Epstein-Barr virus (EBV) called latent membrane protein 1 (LMP1) is a primary contributor to the transformation of human B cells by the virus and the development of EBV-associated B cell malignancies by recruiting signaling molecules provided by the host. One such molecule, the cellular adapter protein TRADD, is among the few direct interaction partners of LMP1. But because TRADD promotes cell death (apoptosis) in the cellular tumor necrosis factor-receptor 1 (TNFR1) signaling pathway, it seems counterintuitive that TRADD could play a role in LMP1 biology, since LMP1 promotes cell survival and proliferation. We provide genetic evidence that TRADD is critical for LMP1 to assemble its transforming signaling network. LMP1 requires TRADD to recruit and activate I-κB kinase β and, thus, to induce canonical NF-κB signaling. Simultaneously, LMP1 masks TRADD's pro-apoptotic activity. We show that LMP1 carries a unique and autonomous viral TRADD-binding domain, which dictates an unusual structure of the LMP1-TRADD complex and the nonapoptotic phenotype of TRADD signaling, irrespective of the receptor context in which this domain is located. Thus, DNA tumor viruses alter the functional properties of cellular signaling molecules to exploit them for their own purpose of cell transformation.

## Abstract 3

A unique Epstein Barr virus-derived protein interaction domain uses the cellular death domain protein TRADD to assemble its transforming signaling complex and dictates a transferable nonapoptitic phenotype of TRADD signaling.

## Introduction

Latent membrane protein 1 (LMP1) is the primary oncogene of Epstein-Barr virus (EBV), which is a human DNA tumor virus of the gamma-herpes virus family that preferentially infects and transforms human B lymphocytes [1]. LMP1 is critical for B lymphocyte transformation by EBV and has oncogenic potential when expressed in the B cell compartment of transgenic mice [1]. Despite the fact that LMP1 promotes cell survival and proliferation, it recruits the pro-apoptotic tumor necrosis factor (TNF)-receptor 1–associated death domain protein (TRADD) [2]. TRADD has been described as the central adapter protein of TNF-receptor 1 (TNFR1), and TRADD mediates TNFR1 induction of apoptosis as well as the activation of NF-κB and c-Jun N-terminal kinase (JNK) [3]. In contrast, the role of TRADD in LMP1 signaling has been unresolved.

LMP1 is a transmembrane protein composed of 386 amino acids (aa) that mimics a constitutively active receptor [4]. Six transmembrane helices mediate the spontaneous formation of LMP1 oligomers in the membrane, which is an essential and sufficient prerequisite to induce signal transduction at the C-terminal cytoplasmic signaling domain (aa 186–386) of the molecule. Two functionally independent regions within the LMP1 signaling domain are involved in B lymphocyte growth transformation and the induction of signal transduction: the C-terminal activating region 1 (CTAR1, aa 194–231) and CTAR2 (aa 351–386) [5]. CTAR1 harbors a P(204)xQxT/S consensus motif, which binds TNFR-associated factors (TRAFs) and induces the noncanonical NF-κB pathway through I-κB kinase α (IKKα) [6–9]. CTAR2 triggers the canonical NF-κB pathway via IKKβ followed by phosphorylation and degradation of I-κB [8,10]. Although IKKβ has an indirect role in CTAR1 function, most likely by up-regulating the expression of critical factors for noncanonical NF-κB signaling, its kinase activity is primarily activated by CTAR2 [8,9,11]. IKKα rather acts as a negative regulator of CTAR2-induced NF-κB activity, whereas IKKγ is required for some but not all canonical CTAR2 signaling [8–10]. The JNK pathway is generally triggered at CTAR2, although CTAR1 can also contribute to JNK activation in some cell lines [12–17]. The motif P(379)VQLSY(384) of CTAR2 is essential for activation of NF-κB and JNK [15,18]. Studies in TRAF-deficient mouse cells suggested critical roles of TRAF3 and TRAF6 in CTAR2 signaling to NF-κB and JNK [10,11,19,20], but it is still unclear how CTAR2 assembles its signaling complex. In contrast to CTAR1, TRAF molecules have no affinity for CTAR2. There is no experimental evidence for a direct or indirect physical interaction of CTAR2 with TRAF3 [5], but TRAF6 translocates to sites of active LMP1, and its indirect interaction with CTAR2 might involve the transcriptional corepressor BS69 as a mediator [19,21].

During the search of CTAR2-binding proteins, TRADD has been pulled out from a yeast–two-hybrid screen using CTAR2 as a bait [2]. Early experiments have indicated a potential role for TRADD in LMP1 signaling. Overexpression of TRADD potentiated NF-κB activation by LMP1 in HEK293 epithelial cells, and co-expression of TRADD harboring a mutated death domain interfered with LMP1 signaling to NF-κB in the same cell line [2,15]. A role for TRADD in CTAR2-triggered JNK activation has also been postulated [14]. However, it cannot be excluded that TRADD overexpression caused unspecific effects unrelated to LMP1 signaling in these studies. More recent experiments showed no significant effect of RNAi-mediated TRADD down-regulation on CTAR2-induced NF-κB and JNK activity in HEK293 cells [11,19]. Therefore, it is currently believed that TRADD is dispensable for LMP1 signaling, and no molecular role of TRADD in organizing the CTAR2 signaling complex has been demonstrated. In analogy to TNFR1 signaling, it has been suggested that TRADD recruits TRAF2 to CTAR2 [14]. However, no experimental evidence for a physical interaction of TRAF2 with the signaling complex at CTAR2 is available. Recent data from TRAF2-deficient cell lines and RNAi experiments showed that TRAF2 is fully dispensable for activation of NF-κB and JNK by LMP1-CTAR2 [10,19,20]. Until today, no definite evidence for a role of TRADD in LMP1 signaling has been provided due to the lack of TRADD-deficient animals or cell lines.

Upon activation with the pro-inflammatory cytokine TNFα, TNFR1 recruits TRADD via direct interaction between the death domains of both molecules [22]. TRADD is believed to serve as a platform for the binding of TRAF2 and RIP1 to set up the TNFR1 complex at the plasma membrane, inducing JNK and NF-κB [23]. Subsequently, TRADD binds the Fas-associated death domain protein (FADD) to activate caspase 8–dependent apoptosis. In contrast to TNFR1, LMP1 is devoid of a death domain, and TRADD's death domain is dispensable for TRADD interaction with LMP1, suggesting that the molecular architectures of the TNFR1-TRADD and LMP1-TRADD complexes are different [15]. However, it is unknown whether this unique type of TRADD recruitment is determined by LMP1 and whether it has relevance for the biological function of LMP1. Although amino acid residues Y384 and Y385 of CTAR2 are known to be essential for TRADD recruitment [2], the functional TRADD-binding site of LMP1 has not been well defined.

Here, we report the first genetic knockout, to our knowledge, of the *TRADD* gene, which we have generated in human B lymphocytes to characterize TRADD's role in LMP1 signal transduction. Using TRADD-deficient cells, we found that TRADD has an essential role in LMP1 activation of canonical NF-κB signaling by mediating the recruitment and activation of IKKβ. In contrast, JNK induction by LMP1 is independent of TRADD, demonstrating that the NF-κB and JNK pathways bifurcate upstream of TRADD at the level of CTAR2. We narrowed down the functional TRADD-binding domain of LMP1 to the 16 C-terminal amino acids of LMP1. The deletion of this domain resulted in a loss of IKKβ binding to LMP1. We were able to transplant the nonapoptotic phenotype of LMP1-induced TRADD signaling to TNFR1 by replacing its death domain by the TRADD-binding sequence of LMP1. Thus, the viral oncoprotein LMP1 exploits TRADD for nonapoptotic signaling, which is intrinsically encoded by the viral TRADD-binding domain but not by the receptor context in which it operates.

## Results

### Somatic Knockout of *TRADD* in Human B Lymphocytes

**Figure 1:**
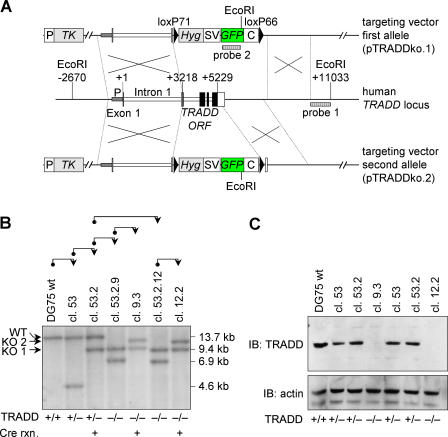
*TRADD* Gene Targeting in Human DG75 B Lymphocytes (A) Knockout strategy. Given base positions relate to the transcriptional start site at position +1. The complete *TRADD* open reading frame (black boxes, exons 2–5 with ATG at position +3218) was deleted by homologous recombination with pTRADDko.1 (first allele) and pTRADDko.2 (second allele). C, CMV promoter; P, promoter; SV, SV40 promoter; TK, thymidine kinase. (B) Southern blot analysis of the resulting DG75 clones with EcoRI-digested chromosomal DNA and the external probe 1. The genealogy of clones is illustrated by arrows. WT, *TRADD* wild-type allele; KO, targeted and Cre-deleted alleles 1 or 2, respectively. (C) TRADD protein expression. Immunoblot analysis using the mouse anti-TRADD antibody.

Studies on the role of TRADD in LMP1 signaling were hampered by the lack of TRADD-deficient mice or cell lines. To solve this problem, we chose to delete the *TRADD* gene from human B lymphocytes, which are the primary target cells of EBV and, thus, the natural compartment of LMP1 expression. The EBV-negative human B cell lines DG75, BJAB, and BL2 were selected as candidates for the *TRADD* knockout due to their diploidy of the *TRADD* locus (unpublished data). We cloned the human *TRADD* gene from a genomic blood cell DNA library in order to construct the two gene targeting vectors pTRADDko.1 (first *TRADD* allele) and pTRADDko.2 (second *TRADD* allele) for the consecutive deletion of both *TRADD* alleles from human somatic cells (Figure 1A and Materials and Methods). Both constructs differed in their 3′ arms homologous to the *TRADD* gene to prevent recombination with the already disrupted *TRADD* allele while targeting the second allele. In DG75 cells, each step of the gene targeting procedure was verified by Southern blotting, resulting in DG75 *TRADD–*/– clones with no encoding TRADD sequences left in the genome (Figure 1B). In contrast to DG75 cells, BJAB and BL2 cells did not support homologous recombination of the targeting vectors with the *TRADD* locus at a detectable frequency (Table S1). The targeting efficiency in DG75 cells was approximately one out of 50 GFP-positive, hygromycin B- and ganciclovir-resistant clones for both *TRADD* alleles (Table S1). As expected, no TRADD protein was expressed in the DG75 *TRADD–*/– clones (Figure 1C). DG75 *TRADD*–/– cells were fully viable and showed similar growth properties to wild-type cells at normal cell densities.

### TRADD Mediates IKKβ Activation by LMP1

**Figure 2:**
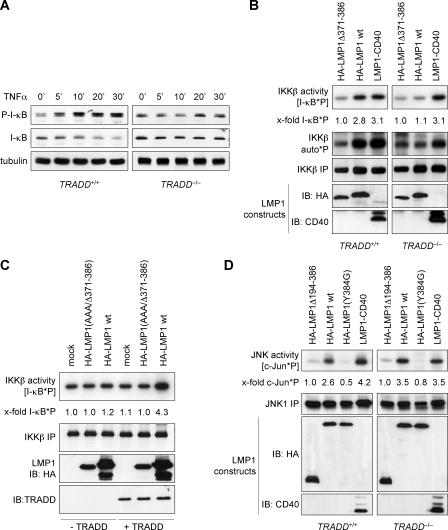
TRADD Is Essential for IKKβ Activation by LMP1, but It Is Dispensable for JNK Induction (A) The knockout of TRADD in DG75 B lymphocytes abolishes TNFR1 activation of the NF-κB pathway. DG75 *TRADD*+/+ or *TRADD*–/– cells were stimulated with 200 ng ml^−1^ soluble human TNFα (Roche) for the indicated times. Levels of phospho-I-κB and I-κB were analyzed on immunoblots. αTubulin served as the loading control. (B) LMP1 activation of IKKβ requires TRADD. DG75 *TRADD*+/+ or *TRADD*–/– cells were electroporated with the indicated LMP1 constructs and Flag-IKKβ. Flag-IKKβ activity was monitored in immunocomplex kinase assays. GST-I-κBα phosphorylation was quantified by a phosphoimager and is given as x-fold induction. Immunoprecipitated Flag-IKKβ and the expressed HA-LMP1 constructs were detected using the anti-Flag (M2) and anti-HA (12CA5) antibodies, respectively. LMP1-CD40 was visualized by the anti-CD40 antibody. IB, immunoblot; IP, immunoprecipitation. (C) Ectopic TRADD expression rescues LMP1 activation of IKKβ in TRADD–/– cells. IKKβ assays were performed in DG75 TRADD–/– cells as in (B). Where indicated, TRADD was expressed at physiological levels by co-transfecting 1 μg of pACYC184-1012.4, which carries the complete human *TRADD* gene including the *TRADD* promoter. (D) JNK1 activation by LMP1 is independent of TRADD. HA-JNK1 immunocomplex kinase assays in DG75 *TRADD*+/+ and *TRADD*–/– cells are shown. Immunoprecipitated HA-JNK1 was dectected with the anti-JNK1 (C17) antibody.

TRADD overexpression and RNAi studies have suggested that TRADD mediates TNFR1 activation of the NF-κB pathway [22,24]. To demonstrate that DG75 *TRADD–*/– cells are suitable tools to study the role of TRADD in signal transduction, we stimulated *TRADD*+/+ and *TRADD–*/– cells with soluble human TNFα and monitored the activity of the NF-κB pathway (Figure 2A). DG75 wild-type cells responded to TNFα stimulation with a rapid phosphorylation and subsequent degradation of I-κB. In contrast, the knockout of TRADD completely abolished TNFα-induced I-κB phosphorylation. This result delivered genetic evidence for an essential role of TRADD in TNFR1 signaling to NF-κB and showed that canonical NF-κB signaling is intact in DG75 cells.

 TRADD interacts with the CTAR2 domain of LMP1, which signals through IKKβ to activate the I-κB–dependent NF-κB pathway [8,10,11]. To investigate a potential role of TRADD in LMP1-induced NF-κB signaling, we performed IKKβ kinase assays in DG75 *TRADD*+/+ and *TRADD–*/– cells, because IKKβ activity is the most proximal and specific readout of CTAR2 signaling on the NF-κB axis (Figure 2B). Due to the high endogenous NF-κB activity levels in DG75 cells, NF-κB reporter gene assays did not result in reliable induction levels after LMP1 expression (unpublished data). HA-LMP1Δ371–386, which lacks the 16 C-terminal amino acids of LMP1, is defective in CTAR2 signaling and served as null control. Expression of wild-type HA-LMP1 resulted in a 2.8-fold activation of IKKβ in *TRADD*+/+ cells, monitored as I-κBα substrate phosphorylation and IKKβ autophosphorylation (Figure 2B). Thus, CTAR2 triggered canonical NF-κB signaling in wild-type cells. Strikingly, CTAR2 lost its potential to induce IKKβ in *TRADD*–/– cells, which clearly showed that CTAR2 requires TRADD to activate the IKKβ pathway (Figure 2B). To demonstrate specificity of this effect for LMP1, we analyzed CD40 signaling in wild-type and TRADD-deficient cells. CD40 does not recruit TRADD and was therefore anticipated to signal also in the absence of TRADD. Confirming this presumption, a constitutively active chimera composed of the LMP1 transmembrane domain and the CD40 signaling domain, LMP1-CD40, induced IKKβ activity in *TRADD*+/+ and *TRADD–*/– cells to similar levels.

To exclude that defects unrelated to TRADD had caused the defective LMP1 signaling in DG75 *TRADD*–/– cells, we tested whether ectopic expression of TRADD restored LMP1 activation of IKKβ in TRADD-deficient cells (Figure 2C). To express TRADD at physiological levels, *TRADD–*/– cells were co-transfected with the pACYC184-1012.4 vector, which carries the complete human *TRADD* gene under the control of its endogenous promoter (see Materials and Methods). Exogenous TRADD expression alone did not induce IKKβ, further confirming that TRADD protein levels lay within a normal range which did not result in TRADD autoactivation. Notably, reintroduction of TRADD enabled LMP1 to activate IKKβ in *TRADD–*/– cells, verifying TRADD's essential role in CTAR2 signaling on the NF-κB axis (Figure 2C). In summary, our results provided strong genetic evidence for a critical role of TRADD in LMP1 signal transduction. In fact, LMP1 requires TRADD to induce the IKKβ/NF-κB pathway.

### NF-κB and JNK Signaling Bifurcate Upstream of TRADD

To further dissect the signaling pathways originating at CTAR2, we tested whether the LMP1-induced JNK pathway required TRADD for its activation. Both HA-LMP1 and LMP1-CD40 readily activated JNK in HA-JNK1 kinase assays in DG75 wild-type cells (Figure 2D). CTAR2 but not CTAR1 triggers JNK activation in DG75 cells, because mutation of Y384 to G within CTAR2 fully abrogated JNK activation as compared with the LMP1Δ194–386 control, which lacks the complete signaling domain. By analyzing JNK signaling in *TRADD*–/– cells, we observed that CD40 activation of JNK1 was independent of TRADD, as expected. Surprisingly, also LMP1 induced JNK1 in *TRADD*–/– cells to levels that were similar to those of wild-type cells (Figure 2D). Thus, activation of the JNK pathway by LMP1 does not involve TRADD, demonstrating that the CTAR2-triggered NF-κB and JNK pathways bifurcate upstream of TRADD at the level of CTAR2.

### TRADD Is Required for IKKβ Interaction with the LMP1 Signaling Complex at CTAR2

In TNFR1 signaling, TRADD mediates TRAF2 and probably also RIP1 binding to the activated signaling complex [3]. Subsequently, the IKK complex is recruited to the receptor through interactions of IKKα/β with TRAF2, and IKKγ with RIP1 [25–27]. Activation of IKKs is achieved through the TAB2/TAK1 complex, which binds to ubiquitinated RIP1 [28]. We show here that TRADD is necessary for IKKβ activation by the CTAR2 domain of LMP1. However, TRAF2/5, RIP1, TAB2, and IKKγ—the key players of TRADD signaling to NF-κB in the TNFR1 context—are all dispensable for LMP1-induced NF-κB activity [10,29]. This raised a question about the molecular function of TRADD in the LMP1 pathway to IKKβ.

**Figure 3:**
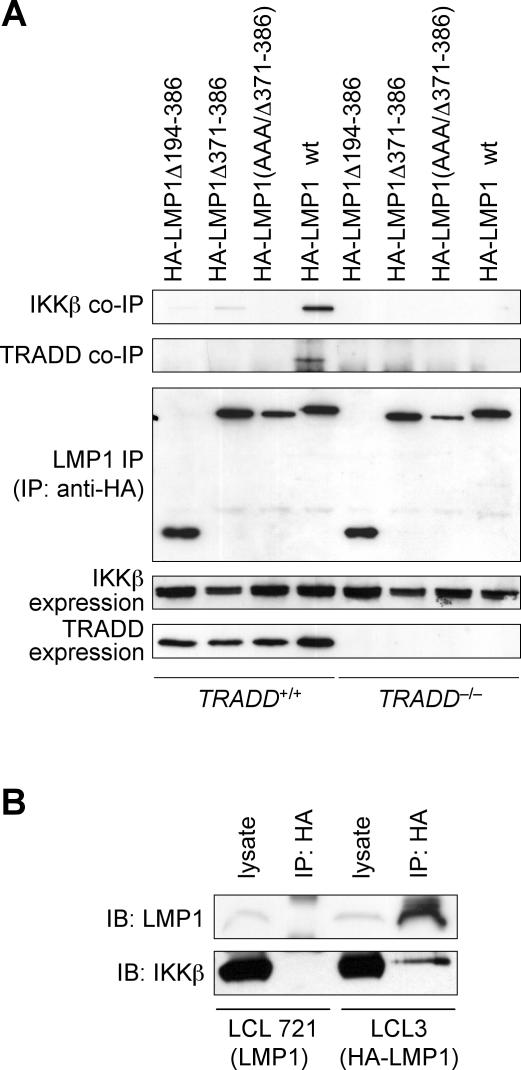
TRADD Mediates the Interaction of IKKβ with CTAR2 (A) DG75 *TRADD*+/+ or *TRADD*–/– cells were electroporated with the indicated HA-LMP1 constructs and Flag-IKKβ. Twenty-four h post transfection, the LMP1 signaling complex was immunoprecipitated using the anti-HA (12CA5) antibody, which was covalently coupled to protein A sepharose beads. Precipitated HA-LMP1 proteins were detected by the anti-HA (12CA5) antibody, and IKKβ and TRADD were detected by the anti-IKKβ and mouse anti-TRADD antibodies, respectively. (B) IKKβ interacts with the LMP1 signaling complex in lymphoblastoid cells. LCL 3 cells were generated by transformation of primary human B cells with a recombinant maxi-EBV, in which the wild-type LMP1 gene had been replaced by HA-tagged LMP1. HA-LMP1 was immunoprecipitated from LCL 3 lysates using anti-HA (12CA5) beads. Parallel immunoprecipitations from LCL 721 cells expressing untagged LMP1 served as a negative control. Precipitated proteins were detected by the mouse anti-LMP1 and the rabbit anti-IKKβ antibodies.

To examine whether LMP1 recruits IKKβ and to evaluate whether TRADD has a role in this process, we performed co-immunoprecipitation assays with HA-tagged LMP1 in DG75 wild-type and TRADD-deficient cells (Figure 3A). IKKβ co-precipitated with HA-LMP1 from *TRADD*+/+ cells. Thus, IKKβ physically interacts with the LMP1 signaling complex. In addition, we found that TRADD was present in the complex together with HA-LMP1 and IKKβ. Deletion of the 16 C-terminal amino acids of LMP1 resulted in a loss of TRADD and IKKβ interaction with LMP1, demonstrating that aa 371–386 of LMP1 are essential for the recruitment of the NF-κB–inducing signaling complex to CTAR2. Remarkably, IKKβ was absent in HA-LMP1 immunoprecipitations from DG75 *TRADD*–/– cells (Figure 3A). These data support an essential role of TRADD in mediating the binding of IKKβ to the LMP1 signaling complex at CTAR2. In contrast to TRADD and IKKβ, we could not detect a physical interaction of TRAF3 or TRAF6 with CTAR2 in our immunoprecipitations from DG75 cells (unpublished data), making it unlikely that both TRAFs have crucial roles in the binding of IKKβ to CTAR2.

Next, we tested whether IKKβ also interacts with LMP1 in EBV-transformed lymphoblastoid cell lines (LCLs) (Figure 3B). For this purpose, primary human B cells were transformed with a recombinant maxi-EBV, which carried HA-LMP1 instead of the wild-type LMP1 gene. The resulting lymphoblastoid cell line LCL 3 endogenously expressed HA-LMP1 at levels comparable to untagged LMP1 of the control LCL 721 (Figure 3B). Endogenous IKKβ co-precipitating with HA-LMP1 was detected in anti-HA immunoprecipitations from HA-LMP1 cells (LCL 3, right panel) but not from LMP1 cells which served as a control for the specificity of anti-HA immunoprecipitation (LCL 721, left panel). These data verified the physical interaction of endogenous IKKβ with the LMP1 signaling complex also in LCLs.

### LMP1 AA 371–386 Comprise a Unique and Transferable TRADD Binding Site

**Figure 5:**
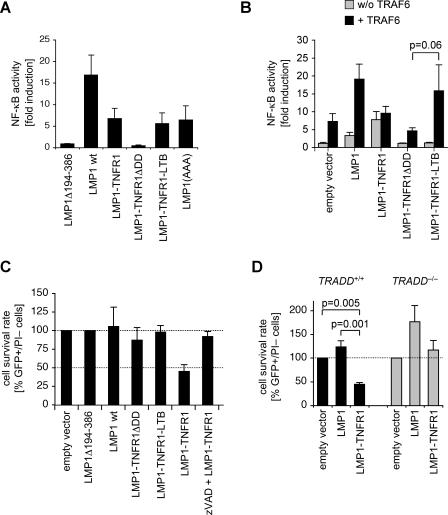
LMP1′s TRADD-Binding Domain (LTB) Induces LMP1-Type Signaling but Does Not Activate Apoptosis in the Context of the TNFR1 Signaling Domain (A) The LTB is functional in the context of the TNFR1 signaling domain. NF-κB reporter assays in HEK293 cells. Data are mean values of three independent experiments ± standard deviation. (B) The transferred LTB determines TRAF6-dependent NF-κB activation. NF-κB reporter assays in TRAF6–/– MEFs are shown. Where indicated (black bars), TRAF6 was co-transfected. Data are mean values of four independent experiments ± standard deviation; statistics: two-tailed Student's *t*-test. (C) Lack of apoptosis induction by LTB in the context of the TNFR1 signaling domain. Transient cell death assays in human BJAB B lymphocytes are shown. The NF-κB pathway was blocked by co-expression of domaint-negative I-κBα(S32/36A). Where indicated, the cells were incubated in the presence of 2 μM zVAD-fmk (zVAD), a pan-caspase inhibitor. PI, propidium iodide. Data are mean values of three independent experiments ± standard deviation. (D) Apoptosis induction by LMP1-TNFR1 is dependent on TRADD. Cell death assays in DG75 *TRADD*+/+ and DG75 *TRADD*–/– cells are shown. Data are mean values of three independent experiments ± standard deviation; statistics: two-tailed Student's *t*-Test.

Here we provide evidence for an active role of TRADD in LMP1 signal transduction. Nevertheless, LMP1 has anti-apoptotic properties, and LMP1 signaling does not trigger cell death, even if the NF-κB pathway is blocked (see: Figure 5) [29]. In contrast, TNFR1 induces caspase-dependent apoptosis through TRADD (see: Figure 5) [24,30]. The TRADD interaction sites of LMP1 and TNFR1 are not related, because LMP1 has no death domain to bind TRADD. Based on these observations, we asked whether the unique TRADD-binding site of LMP1 might determine the non-apoptotic phenotype of virus-induced TRADD signaling. Alternatively, the receptor context of LMP1′s TRADD-binding domain might dictate the outcome of TRADD signaling. To answer this question, we transferred LMP1′s TRADD-binding domain into the context of the TNFR1 signaling domain and evaluated the apoptotic potential of the resulting chimera.

**Figure 4:**
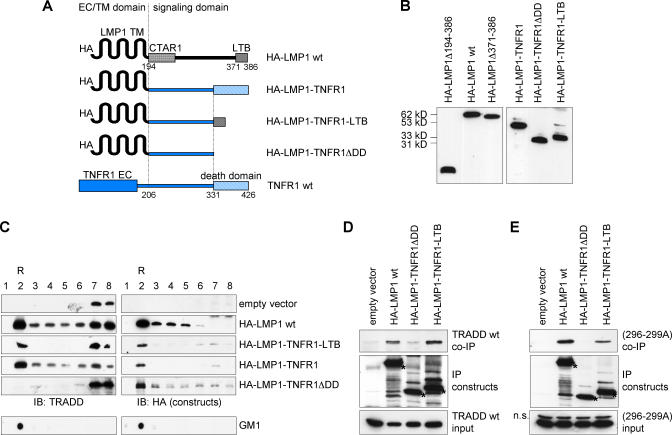
Amino Acids 371–386 of LMP1 Encompass the Functional TRADD-Binding Domain (A) Domain swapping constructs. HA-LMP1-TNFR1 is a chimera of the LMP1 transmembrane domain and the signaling domain of TNFR1. HA-LMP1-TNFR1ΔDD lacks the TNFR1 death domain (DD). HA-LMP1-TNFR1-LTB carries aa 371–386 of LMP1 instead of the TNFR1 death domain. EC, extracellular domain; LTB, LMP1 TRADD-binding domain; TM, transmembrane domain; wt, wild type. (B) Expression in HEK293 cells. Cells were lysed in the presence of 0.1% NP40, and proteins were detected on immunoblots of total cell lysates by the anti-HA (12CA5) antibody. (C) TRADD recruitment into lipid rafts. HEK293 cells were transfected with the indicated constructs together with expression vectors for TRADD and p35. Twenty-four h post transfection, lipid rafts were isolated. Fraction 2 contains lipid rafts (R), as detected on dot blots by the raft marker GM1. The anti-TRADD (H278) and anti-HA (12CA5) antibodies were used to visualize TRADD and HA-tagged constructs on immunoblots, respectively. (D) Replacing the TNFR1 death domain, aa 371–386 of LMP1 are sufficient to recruit TRADD to the TNFR1 signaling domain. HEK293 cells were transfected with the indicated constructs together with expression vectors for TRADD wild type and p35. The HA-tagged constructs (asterisks) were immunoprecipitated via HA and detected by the anti-HA (12CA5) antibody. The mouse anti-TRADD antibody was used to stain TRADD. IP, immunoprecipitation; wt, wild type. (E) Interaction of LTB with TRADD is independent of a functional TRADD death domain. TRADD(296–299A) was co-transfected together with the indicated contructs. TRADD(296–299A) was detected by the anti-TRADD (H278) antibody. n.s., non-specific band.

Amino acids 371–386 of LMP1 were essential for TRADD-binding (Figure 3A). We speculated that this motif might also be sufficient for TRADD interaction and, thus, contains the functional LMP1 TRADD-binding site (LTB). Therefore, we replaced the TNFR1 death domain of the HA-tagged LMP1-TNFR1 chimera by LMP1 aa 371–386, resulting in the construct HA-LMP1-TNFR1-LTB (Figure 4A). Due to spontaneous oligomerization of the LMP1 transmembrane part of the molecule, HA-LMP1-TNFR1 and its derivatives induce constitutive signaling specified by the type of fused signaling domain [4,31]. Thereby, all chimeras based upon HA-LMP1-TNFR1 are independent of a ligand and their signaling readouts can be directly compared with HA-LMP1. HA-LMP1, HA-LMP1-TNFR1, and all derivatives were expressed to similar levels in HEK293 cells (Figure 4B).

First, it was important to show that aa 371–386 of LMP1 were sufficient to mediate TRADD interaction. Both, LMP1 and TNFR1 partially localize to, and signal from, membrane lipid rafts [23,32,33]. Therefore, we tested if HA-LMP1-TNFR1-LTB recruited TRADD into lipid rafts in HEK293 cells. A substantial fraction of HA-LMP1, HA-LMP1-TNFR1, and its derivatives localized to the lipid raft fraction (Figure 4C). Because of a reduced solubility of HA-LMP1-TNFR1 in Triton X-100 (unpublished data), this assay delivered qualitative rather than quantitative data regarding the amounts of expressed HA-LMP1-TNFR1 and its derivatives. HA-LMP1 recruited TRADD to rafts. Also HA-LMP1-TNFR1 relocated a major fraction of TRADD into this signaling-active compartment of the membrane. The deletion of the TNFR1 death domain completely abolished the ability of HA-LMP1-TNFR1 to interact with TRADD, as was shown by the absent shift of TRADD into the raft fraction in the presence of HA-LMP1-TNFR1ΔDD. From this observation, we also concluded that aa 206–331 of the TNFR1 signaling domain were not sufficient to mediate TRADD binding to TNFR1. When we replaced the death domain of TNFR1 by aa 371–386 of LMP1, the resulting HA-LMP1-TNFR1-LTB chimera efficiently recruited TRADD into lipid rafts (Figure 4C). This result showed that the 16 C-terminal amino acids of LMP1 are not only essential but also sufficient for TRADD interaction and, thus, comprise the functional TRADD-binding domain of LMP1.

To further substantiate our findings, we performed immunoprecipitation assays in HEK293 cells. Wild-type TRADD co-precipitated with HA-LMP1, whereas HA-LMP1-TNFR1ΔDD failed to pull down TRADD (Figure 4D). Confirming aa 371–386 as the self-sufficient TRADD-binding site of LMP1, TRADD bound to HA-LMP1-TNFR1-LTB and HA-LMP1 equally well (Figure 4D).

Mutation of aa 296–299 to alanines in TRADD's death domain strongly impaired TRADD binding to TNFR1 as well as TRADD activation of NF-κB, but had no negative effect on TRADD's interaction with LMP1 in glutathione S-transferase (GST) pulldown assays [15,34]. To evaluate whether this unique type of interaction between LMP1 and TRADD is encoded by the TRADD-binding site of LMP1, we tested if TRADD (296–299A) interacted with HA-LMP1-TNFR-LTB in immunoprecipitations (Figure 4E). HA-LMP1 efficiently bound TRADD (296–299A). After its transfer into the context of the TNFR1 signaling domain, the TRADD-binding site of LMP1 was still able to recruit TRADD (296–299A), albeit to a somewhat lesser extend than wild-type LMP1 (Figure 4E). Taken together, these experiments showed that aa 371–386 of LMP1 compose an autonomous viral TRADD-binding domain, which determines the unique type of interaction with TRADD, irrespective of the neighboring receptor context.

### The Viral TRADD Binding Site of LMP1 Determines Nonapoptotic TRADD Signaling

Next, we tested if the TRADD-binding site of LMP1 not only recruited TRADD when replacing the death domain of TNFR1, but also induced signal transduction (Figure 5A). Expression of wild-type HA-LMP1 caused a 17.0-fold induction of NF-κB activity in HEK293 cells. The contribution of CTAR2 was a 6.4-fold NF-κB activation, as determined by transfection of HA-LMP1(AAA), which carries an inactivating P(204)xQxT to AxAxA mutation in its CTAR1 domain. The HA-LMP1-TNFR1-LTB chimera also activated NF-κB to levels that were comparable to HA-LMP1(AAA), demonstrating that aa 371–386 of LMP1 retained their full competence to induce signal transduction after the transfer to the TNFR1 signaling domain. In addition, also JNK1 was activated by the HA-LMP1-TNFR1-LTB chimera, further confirming the functionality of LTB in the HA-LMP1-TNFR1-LTB chimera (Figure S1). The TRADD-binding site of LMP1 induced similar NF-κB levels as the TNFR1 death domain, demonstrating that both TRADD-binding domains are equally efficient with respect to NF-κB activation (Figure 5A).

In contrast to TNFR1, LMP1 signaling to NF-κB is dependent on TRAF6 [10,11,21]. To show that LMP1 aa 371–386 induced LMP1-type signaling in the context of the TNFR1 signaling domain, we evaluated if HA-LMP1-TNFR1-LTB activation of NF-κB is dependent on TRAF6. In contrast to HA-LMP1-TNFR1, the HA-LMP1-TNFR1-LTB chimera indeed required the reconstitution of TRAF6 expression in *TRAF6*–/– mouse embryonic fibroblasts (MEFs) to induce NF-κB (Figure 5B). Taken together, these results indicate that the TRADD-binding site of LMP1 was fully functional in the context of the TNFR1 signaling domain and encoded an LMP1-type signaling and interaction with TRADD.

Finally, we investigated whether the TRADD-binding site of LMP1 had the potential to induce cell death in the context of the TNFR1 signaling domain. Cell death assays in human BJAB B lymphocytes showed that expression of wild-type HA-LMP1 did not interfere with cell survival in the absence of NF-κB signaling, which was inhibited by co-transfection of dominant-negative I-κBα(S32/36A) (Figure 5C). In contrast, TNFR1 signaling induced by HA-LMP1-TNFR1 strongly reduced the survival rate of BJAB cells to 45.3 % of the controls. Cell death induced by HA-LMP1-TNFR1 was caspase-dependent, because the pan-caspase inhibitor zVAD-fmk fully rescued cell survival after HA-LMP1-TNFR1 expression (Figure 5C). Moreover, HA-LMP1-TNFR1 required TRADD to exert its killing activity in B lymphocytes (Figure 5D). Because TNFR1 has been demonstrated to induce apoptosis specifically through a TRADD- and caspase-dependent pathway [24], HA-LMP1-TNFR1–induced cell death is most likely due to apoptosis. Most notably and in contrast to HA-LMP1-TNFR1, the HA-LMP1-TNFR1-LTB chimera did not induce cell death (Figure 5C). Thus, although being fully functional with regard to the binding of TRADD and the induction of signal transduction, the TRADD-binding site of LMP1 had no apoptotic potential in the context of the TNFR1 signaling domain. We concluded from these results that the unique TRADD-binding site of LMP1, but not the context of the receptor signaling domain, encodes the nonapoptotic type of viral TRADD signaling.

### The TRADD-Binding Site of LMP1 Converts TNFR1 into a Nonapoptotic Receptor

**Figure 6:**
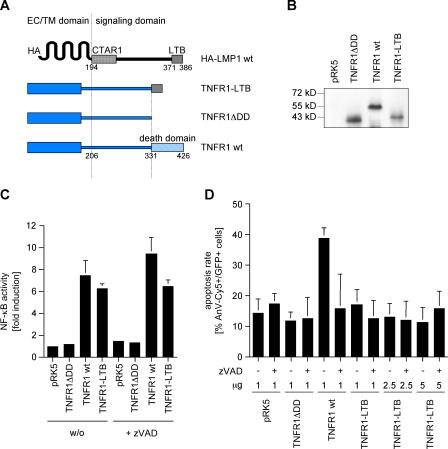
TNFR1 Is Converted into a Nonapoptotic Receptor upon Replacement of Its Death Domain by the TRADD-Binding Site of LMP1 (A) Schematic depiction of the chimeras. (B) Ectopic expression of the indicated constructs in TNFR1/TNFR2 double-negative MEFs, detected by the anti-TNFR1 (H5) antibody. (C) Both TNFR1 wild type and TNFR1-LTB induce NF-κB. NF-κB reporter assays in TNFR1/TNFR2 double-negative MEFs are shown. Data have been corrected for protein expression levels of TNFR1 constructs and are mean values of three independent experiments ± standard deviation. (D) TNFR1, but not TNFR1-LTB, induces apoptosis. TNFR1/TNFR2 double-negative MEFs were electroporated with the indicated amounts of constructs together with GFP. Five h post transfection, the cells were stained with Annexin V-Cy5 (AnV-Cy5) and analyzed by flow cytometry. AnV-Cy5+/GFP+ staining indicated apoptosis induced by the transfected constructs. In (C) and (D), the cells were incubated with 2 μM zVAD-fmk (zVAD) throughout the experiment where indicated.

To further corroborate our findings, we transferred LMP1 aa 371–386 into the context of wild-type TNFR1, replacing TNFR1′s death domain (Figure 6A). The resulting chimera, TNFR1-LTB, was devoid of any other LMP1 sequences except of LMP1′s TRADD-binding site. TNFR1ΔDD lacking its death domain served as a control. After electroporation, all constructs were expressed to similar levels in TNFR1/TNFR2-double deficient MEFs (Figure 6B). We used TNFR-deficient cells for our experiments to avoid cross-talk or the formation of heterocomplexes between the exogenously expressed TNFR1-LTB chimera and endogenous wild-type TNF-receptors. Activation of the TNFR1 constructs was achieved by spontaneous patching upon high exogenous expression in TNFR-deficient cells. Both, TNFR1 and TNFR1-LTB induced NF-κB to comparable levels in TNFR-deficient MEFs in the presence or absence of caspase inhibition (Figure 6C). Thus, the TRADD-binding domain of LMP1 was functional also in the context of wild-type TNFR1. However, whereas TNFR1 expression resulted in a massive induction of caspase-dependent apoptosis in TNFR-deficient MEFs, TNFR1-LTB did not affect viability, even if TNFR1-LTB was transfected at 5-fold higher DNA concentrations than wild-type TNFR1 (Figure 6D). These results confirmed our conclusions that aa 371–386 of LMP1 make up a self-sufficient, viral TRADD-binding domain which encodes a unique and nonapoptotic type of TRADD signaling.

## Discussion

Our results provide definite evidence that the cellular pro-apoptotic TRADD protein is a critical signaling mediator of the EBV oncoprotein LMP1, and they show that DNA tumor viruses have developed means to modulate the molecular and functional properties of cellular signaling molecules. We have demonstrated that EBV masks TRADD's pro-apoptotic activity and that this unique viral function is intrinsically encoded by the TRADD-binding domain of the LMP1 molecule. Moreover, this property of the 16 C-terminal amino acids of LMP1 is transferable to other receptors such as TNFR1. EBV exploits TRADD for NF-κB signaling by LMP1-CTAR2, which contributes important growth factor-like signals for efficient proliferation of EBV-transformed B lymphocytes [35,36]. Quantitative analysis has revealed that a recombinant maxi-EBV harboring a LMP1(Y384G) mutant has a ∼90% reduced potential of inducing long-term B cell proliferation compared to wild-type virus [37]. LMP1 also transforms rat fibroblasts in culture [38]. Whereas CTAR1 but not CTAR2 was the essential domain for in vitro transformation of Rat1 cells in one report [39], other studies supported a critical role of CTAR2 in oncogenic transformation of rat fibroblasts in vitro as well as in vivo [40,41]. Notably, tumor formation by LMP1-transformed Rat1 cells in nude mice essentially requires NF-κB activity and a functional CTAR2 domain [41]. According to our data, it is therefore highly likely that TRADD is critically involved in mediating these functions of CTAR2, extending the biological spectrum of TRADD activity to cell proliferation and transformation.

So far, studies on the role of TRADD in LMP1 signaling were solely based on the overexpression of TRADD and TRADD mutants, or RNAi-mediated knockdown of TRADD [2,11,14,15,19]. Such experiments yielded conflicting results with respect to the potential role of TRADD in LMP1 signal transduction (see Introduction). The knockdown of TRADD in HEK293 epithelial cells even suggested that TRADD is fully dispensable for LMP1 signaling [11,19], although residual TRADD protein might have rescued pathway activities in RNAi experiments. Alternatively, this result might reflect different cell type–specific functions of TRADD. To be able to investigate TRADD's molecular role in LMP1 signaling in a clean genetic system, we generated a *TRADD* knockout by deleting both *TRADD* alleles from human DG75 B lymphocytes. This is a biologically relevant cell system, because human B lymphocytes are the target cells of EBV. DG75 cells also express TNFR1 and readily responded to TNFα stimulation with activation of the NF-κB pathway. The knockout of *TRADD* abrogated TNFα-mediated NF-κB activation in DG75 cells, demonstrating that TNFR1 activation of the canonical NF-κB pathway critically depends on TRADD and that TRADD signaling is intact in DG75 cells.

The molecular mechanisms by which CTAR2 of LMP1 activates NF-κB are poorly understood. It has been unclear which direct interaction partners of CTAR2 mediate NF-κB activation. TRADD was believed to be dispensable for LMP1 signaling to NF-κB [11]. TRAF3 and TRAF6 are required for NF-κB activation by CTAR2, but TRAF molecules do not interact with CTAR2 directly [5]. In addition, knockdown of BS69, a potential mediator of TRAF6 binding to CTAR2, did not affect LMP1 signaling to NF-κB [42]. Here we demonstrate that the CTAR2 domain of LMP1 recruits IKKβ for signaling and that IKKβ recruitment and, thus, activation by LMP1 depends on TRADD. In TNFR1 signaling, activation of IKKβ can be functionally separated from its recruitment to the signaling complex. IKKβ directly binds to TRAF2, which gets recruited to TNFR1 via TRADD [25,26,30]. The activation of IKKβ requires the interaction of IKKγ, the regulatory subunit of the IKK complex, with ubiquitinated RIP1 [28]. In contrast, TRAF2, RIP1, and IKKγ are dispensable for LMP1 signaling [10,29].

**Figure 7:**
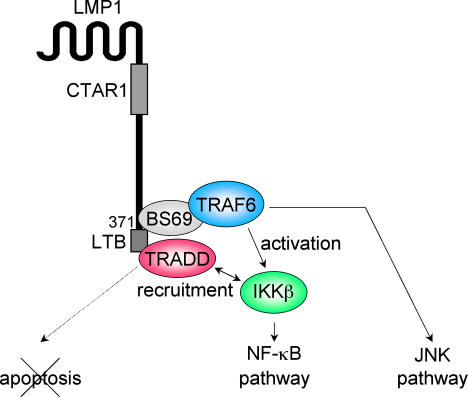
Schematic Model of TRADD's Role in Nonapoptotic Signaling Induced by the TRADD-Binding Domain of LMP1 The unique interaction of the 16 C-terminal amino acids of LMP1 (LTB) with TRADD prevents apoptosis induction through TRADD, which is an intrinsic and transferable function of LTB. Additional LMP1 sequences are not required for masking TRADD's apoptotic activity. TRADD mediates the recruitment of IKKβ to the LMP1 signaling complex through a yet unidentified factor. Activation of IKKβ requires a parallel pathway that is dependent on TRAF6. TRAF6 activation of IKKβ most likely involves TAK1 and TAB1 (not depicted). The JNK pathway is TRADD-independent and works through TRAF6 and, probably, BS69. To keep this model concise, only key molecules of CTAR2 signaling are shown. Refer to the text for more details.

How does NF-κB signaling at CTAR2 work? We propose a mechanism of IKKβ activation at CTAR2 that involves two pathways: a TRADD-dependent IKKβ recruitment pathway and a parallel activation pathway that is critically mediated by TRAF6 (Figure 7). Such a mechanism resolves the paradox that TRADD is solely required for CTAR2-triggered NF-κB signaling, whereas TRAF6 is essential for both NF-κB and JNK activation. Because TRAF2 is dispensable for NF-κB induction by LMP1 [10], a yet-undefined factor must be postulated that bridges IKKβ binding to TRADD in LMP1 signaling. We exclude TRAF3 or TRAF6 as candidates, because TRADD has affinity for only TRAF1 and TRAF2 [43], and we did not detect TRAF3 or TRAF6 in immunoprecipitations together with TRADD and IKKβ (unpublished data). Potential candidates might include p62/PKCζ [44]. Despite their role in canonical NF-κB signaling, a physical and/or functional interaction of TRAF3 and TRAF6 with CTAR2 must be independent of TRADD, because both TRAFs are critical for JNK activation by CTAR2, which is still intact in TRADD–/– cells. The transcriptional corepressor BS69 was suggested as the potential mediator of TRAF6 interaction with CTAR2 [42], although a recent report showed that endogenous BS69 is exclusively located within the nucleus interacting with chromatin [45]. However, cytoplasmic BS69 protein levels beyond the detection limit of the applied BS69 antibody possibly suffice to mediate LMP1 signaling. It is also conceivable that additional and yet-unknown factors might be involved in mediating the interaction of TRAFs with CTAR2.

Upon overexpression, TRADD is a highly apoptotic protein [22]. In TNFR1 signaling, TRADD mediates the activation of caspase-dependent apoptosis through FADD [22,24,30]. In contrast, LMP1 is a transforming protein with anti-apoptotic properties that does not induce programmed cell death, even if NF-κB signaling is blocked (Figure 5) [5,29]. We showed here that TRADD is essential for NF-κB induction by CTAR2, a domain that delivers signals critical for long-term proliferation of EBV-transformed B lymphocytes [35,37]. Thus, TRADD has a unique biological role in LMP1-induced cell proliferation and, therefore, contributes to B cell transformation by EBV. How does LMP1 achieve to exploit TRADD without inducing apoptosis? Our results demonstrated that aa 371–386 of LMP1 encompass an autonomous and unique viral TRADD-binding domain that encodes the nonapoptotic properties of TRADD signaling. This domain has no sequence homology to any known TRADD-binding site of cellular receptors or signaling molecules. Accordingly, the interaction between LMP1 and TRADD does not require a functional death domain in either of the two molecules. Our data demonstrated that this unique molecular structure of the LMP1–TRADD complex is intrinsically determined by the TRADD-binding site of LMP1 and can be transferred together with the nonapoptotic phenotype of TRADD signaling to TNFR1. Therefore, the unique interaction of TRADD with the TRADD-binding site of LMP1 must prevent apoptosis induction by TRADD. Accordingly, we could not detect an interaction of TRADD with the apoptosis mediator FADD in the presence of LMP1 (unpublished data). Sequences within the TRADD death domain, which are required for apoptosis induction, might be masked by components of the LMP1–TRADD complex. Based on our domain-swapping experiments, we can further exclude the possibility that signals originating elsewhere at the LMP1 signaling domain, for instance at CTAR1, are required to suppress apoptosis induction by TRADD, because the 16 C-terminal amino acids of LMP1 did not induce apoptosis in the context of wild-type TNFR1.

We also showed that LMP1 and its anticipated functional analogue, CD40, substantially differ in their receptor-proximal mechanisms activating signal transduction. Very much in contrast to LMP1, CD40 does not require TRADD for signaling. Thus, although both molecules share similarities regarding their role in supporting B-cell proliferation [46], they work through different receptor-proximal signaling molecules. This is in line with previous observations that LMP1 assembles a more efficient signaling complex than CD40 and that LMP1, but not CD40, signaling is dependent on TRAF3 [20,33,47].

In summary, our experiments defined an essential and unique role for TRADD in signaling of the viral oncoprotein LMP1. The viral TRADD-binding site of LMP1 determines the nonapoptotic and TRAF6-dependent type of TRADD signaling which leads to the activation of NF-κB. Thus, the human DNA tumor virus EBV has developed strategies to alter the functional and molecular properties of cellular signaling molecules to exploit them for its own purpose of cell transformation.

## Materials and Methods

### Plasmids and cloning.

pHEBO empty vector, pCMV-HA-LMP1 based on pHEBO, pSV-LMP1-CD40, pcDNA3-p35, pRK5 empty vector, pRK-myc-TRADD, pRK-myc-TRADD(296–299A), pCMV-I-κBα(S32/36A), pcDNA3-Flag-IKKβ, and the NF-κB reporter plasmid 3xκBL have been described previously [15,21,48,49]. pEGFP-C1 is commercially available (BD Clontech). The following expression vectors were cloned on the basis of pCMV-HA-LMP1 by PCR approaches: pCMV-HA-LMP1(AAA), pCMV-HA-LMP1(Y384G), pCMV-HA-LMP1Δ194–386, pCMV-HA-LMP1Δ371–386 and pCMV-HA-LMP1(AAA/Δ371–386). The LMP1-TNFR1 fusion was generated by overlap-extension PCR and cloned into the background of pCMV-HA-LMP1 to obtain pCMV-HA-LMP1-TNFR1. The vectors pCMV-HA-LMP1-TNFR1ΔDD and pCMV-HA-LMP1-TNFR1-LTB were generated by PCR approaches based upon pCMV-HA-LMP1-TNFR1. To generate pRK5-TNFR1, the sequence of human TNFR1 was amplified by PCR from a TNFR1 cDNA (kind gift from H. Wajant) and cloned into pRK5. pRK5-TNFR1ΔDD and pRK5-TNFR1-LTB were cloned by PCR approaches. To generate pRK5-HA-JNK1, human JNK1α1 cDNA (kind gift from M. Karin) was N-terminally fused with a hemagglutinin (HA)-tag by PCR and cloned into pRK5. All constructs were verified by sequencing.

### *TRADD* gene targeting in human B lymphocytes.

The human *TRADD* gene was cloned from the blood cell PAC library RPCI,3–5 (P. de Jong, Roswell Park Cancer Institute). A 13,702-bp EcoRI fragment of the clone RPCIP704M111012 encompassing the complete *TRADD* gene was partially sequenced and subcloned into pACYC184, resulting in the vector pACYC184-1012.4. Based on this *TRADD* clone, we constructed the gene targeting vectors pTRADDko.1 and pTRADDko.2. The 5′ homologous arms of both vectors were identical, whereas the constructs differed in their 3′ arms homologous to the *TRADD* gene to prevent recombination of pTRADDko.2 with the already disrupted *TRADD* allele. 10^7^ DG75 cells were electroporated with 20 μg of linearized pTRADDko.1. The cells were selected in RPMI medium containing 10% fetal calf serum in the presence of 50% cell-free pre-conditioned medium, 400 μg ml^−1^ hygromycin B and 40 μM ganciclovir (Cymeven, Hoffmann-La-Roche) for simultaneous positive and negative selection. Successful gene targeting was verified by Southern blot analysis of EcoRI-digested cellular DNA using the external probe 1 and the internal probe 2. Subsequently, the hygromycin-resistance (Hyg-R) and GFP expression cassettes were removed from the disrupted *TRADD* gene locus by Cre-mediated recombination. Due to the use of the modified loxP66 and loxP71 sites [50], no functional loxP sequence resided in the genome after Cre reaction. To target the remaining wild-type *TRADD* allele, resulting TRADD+/– clones were subjected to a second gene targeting round using the pTRADDko.2 vector.

### Immunoprecipitation, immunoblotting, and antibodies.

10^7^ DG75 cells were electroporated in a BioRad Gene Pulser at 240 V and 975 μF with 5 μg of the indicated LMP1 constructs and 1 μg Flag-IKKβ. Total transfected DNA was adjusted to 18 μg with inert DNA. Five transfection samples were pooled per immunoprecipitation. Twenty-four h post transfection, the cells were lysed in IP-lysis buffer (50 mM HEPES, pH 7.5, 150 mM NaCl, 0.1% NP40, 5 mM EDTA, Roche protease inhibitor cocktail). HA-tagged proteins were immunoprecipitated by the anti-HA (12CA5) antibody (Roche) which had been covalently coupled to protein A sepharose beads (Amersham) using dimethyl pimelimidate (Pierce). After immunoprecipitation, beads were washed three times with IP-lysis buffer and precipitated proteins were analyzed on immunoblots. HEK293 cells were transfected in five 10-cm dishes per immunoprecipitation with 3.5 μg of LMP1 constructs, 0.5 μg of TRADD constructs, and 1.5 μg of pcDNA3-p35 using the polyfect reagent (Qiagen). LCL 3 lymphoblastoid cells were generated by in vitro transformation of primary human B lymphocytes with a recombinant maxi-EBV in which the wild-type LMP1 gene had been replaced by HA-LMP1 as described [37]. HA-LMP1 was immunoprecipitated from LCL 3 lysates using anti-HA (12CA5)-coupled protein A sepharose beads (see above). The LCL 721 [51] expressing untagged LMP1 was used as a control. For standard immunoblotting, cells were lysed in IP-lysis buffer containing 0.1% NP40 as a detergent (see above). The following primary antibodies were used: Actin (I-19), CD40 (C-20), I-κBα (C-21), IKKβ (H-470), JNK1 (C-17), TNFR1 (H-5), TRADD (H-278), αTubulin (B-5-1-2) (all from Santa Cruz Biotech.), HA (12CA5) (Roche), Flag (M2) (Sigma), TRADD (mouse, BD Transduction Lab.), and phospho-I-κBα(Ser32) (New England Biolabs).

### Isolation of membrane lipid rafts.

For lipid raft recruitment assays, HEK293 cells were transfected with the indicated constructs in 10-cm dishes. Twenty-four h post transfection, the cells were lysed in TXNE buffer (25 mM Tris-HCl, pH 7.4, 150 mM NaCl, 5 mM EDTA, 0.5% Triton X-100, Roche protease inhibitor cocktail) at 4 °C and subsequently fractionated in an OptiPrep (Progen) density gradient as described [52]. Membrane lipid rafts were found enriched in the second fraction from top out of eight 500-μl fractions. The raft-specific sphingolipid GM1 served as a raft marker and was detected on dot blots using HRP-coupled cholera toxin subunit B (Sigma).

### Immunocomplex kinase assay.

5 × 10^6^ DG75 cells were electroporated with 5 μg of the indicated constructs together with 3 μg of pRK5-HA-JNK1 for JNK assays or with 2 μg of the LMP1 constructs and 5 μg of pcDNA3-Flag-IKKβ for IKKβ assays. GFP and p35 were co-transfected. To rescue TRADD expression, DG75 *TRADD*–/– cells were co-transfected with 1 μg of pACYC184-1012.4 (see above)**.** Total amounts of transfected DNA were adjusted to 15 μg with inert DNA. As described [21], the cells were lysed 24 h post transfection, HA-JNK1 or Flag-IKKβ was immunoprecipitated by the anti-HA (3F10) or anti-Flag (M2) antibodies, respectively, and immunocomplex kinase assays were performed using purified GST-c-Jun or GST-I-κBα as substrates.

### Cell death assay in B lymphocytes.

3 × 10^6^ B lymphocytes were electroporated in a Bio-Rad gene pulser with 10 μg of the indicated LMP1 constructs together with 1 μg of pEGFP-C1 and 3 μg of pCMV-I-κBα(S32/36A) to block the NF-κB pathway. Twenty-four h post transfection, the cells were stained with propidium iodide (PI), and GFP+/PI– cells were quantified by flow cytometry in a FACSCalibur cytometer (Becton Dickinson) as described [31]. The number of successfully transfected viable cells (GFP+/PI–) in the empty vector reference was set to 100% survival. The reduction of green living cells in a test sample versus the reference was a direct measure for the cell death rate induced by the co-transfected gene [31].

### Apoptosis assay.

10^7^ TNFR1/TNFR2 double-deficient MEFs [53] were electroporated at 240 V and 975 μF in a BioRad Gene Pulser with the given amounts of the indicated TNFR1 constructs together with pEGFP-C1. Total transfected DNA was adjusted to 16 μg with inert DNA. Six h post transfection, the cells were stained with Cy5-labeled annexin V (Biocat) and analyzed for GFP expression and annexin V staining by flow cytometry. AnV-Cy5+/GFP+ cells were indicative for successfully transfected cells undergoing apoptosis.

### Reporter assay.

TRAF6-deficient MEFs [54] and HEK293 cells were transfected in six-well plates with the indicated constructs together with pcDNA3-p35, the NF-κB reporter 3xκBL, and CMVβGal (BD Clontech) using Polyfect (Qiagen). Twenty-four h post transfection, the cells were lysed in luciferase lysis buffer (100 mM KP_i_, pH 7.8, 1 mM DTT, 1% Triton X-100). Firefly luciferase and β-galactosidase were measured as described [21]. 10^7^ TNFR1/TNFR2 double-deficient MEFs were electroporated with the indicated constructs together with the NF-κB reporter 3xκBL and pCMV-RL (Promega). Twenty-four h post transfection, firefly and renilla luciferase activities were measured and analyzed using the Dual-Luciferase reporter assay system (Promega). Luciferase activities were standardized for β-galactosidase or renilla luciferase activities, respectively.

## Supporting Information

Figure S1 The TRADD-binding domain of LMP1 Induces JNK Signaling in the Context of the TNFR1 Signaling Domain HEK293 cells were transfected with the indicated constructs together with HA-JNK1. In addition, the cells were incubated with 2 μM zVAD-fmk to inhibit apoptosis induced by HA-LMP1-TNFR1. Twenty-four h post transfection, HA-JNK1 was immunoprecipitated from cell lysates and HA-JNK1 immunocomplex kinase assays were performed using GST-c-Jun as a substrate. Phosphorylation levels were quantified using a phosphoimager.
(845 KB TIF)

Table S1 Quantitative Results of the *TRADD* Gene Targeting in the EBV-negative B Cell Lines DG75, BJAB, and BL2 (43 KB PDF)

### Accession Numbers

The GenBank (http://www.ncbi.nlm.nih.gov/Genbank/index.html) accession number for human *TRADD* gene is AY995114.

## Abbreviations

aa: amino acids
CTAR: C-terminal activating region
EBV: Epstein-Barr virus
FADD: Fas-associated death domain protein
IKKβ: I-κB kinase β
JNK: c-Jun N-terminal kinase
LCL: lymphoblastoid cell line
LMP1: latent membrane protein 1
LTB: LMP1 TRADD-binding site
MEF: mouse embryonic fibroblast
TNF: tumor necrosis factor
TNFR1: TNF-receptor 1
TRADD: TNF-receptor 1–associated death domain protein
TRAF: TNFR-associated factor
