# Supplementary material for: The Viral Oncoprotein LMP1 Exploits TRADD for Signaling by Masking Its Apoptotic Activity
Source: PLoS Biol. 2008 Jan 15;6(1):e8. doi: 10.1371/journal.pbio.0060008 (PMC2174972; doi:10.1371/journal.pbio.0060008)
Supplement: Supplementary file 1 [file pbio.0060008.st001.pdf]

Table S1

cell line	DG75		BJAB		BL2	
	number of clones tested	clones with successful targeting or recombination	number of clones tested	clones with successful targeting or recombination	number of clones tested	clones with successful targeting or recombination
gene targeting, first allele	145[a]	2	204[a]	0[c]	0[d]	n.a.
Cre-recomb., first allele	27[b]	27	n.a.	n.a.	n.a.	n.a.
gene targeting, second allele	108[a]	2	n.a.	n.a.	n.a.	n.a.
Cre-recomb., second allele	10[b]	7	n.a.	n.a.	n.a.	n.a.

Table S1. Quantitative Results of the *TRADD* Gene Targeting in the EBV-negative B-cell Lines DG75, BJAB and BL2.

a, After each gene targeting round, GFP-expressing and hygromycin B/gancilcovir-resistant clones were tested for successful homologous recombination at the *TRADD* locus by Southern blot analysis.

b, After Cre-reaction, GFP-negative clones were tested for successful deletion of the GFP/hygromycin B cassette from the targeted allele by Southern blot analysis.

c, None of the tested GFP-expressing and hygromycin B/gancilcovir-resistant clones in BJAB cells carried a targeted *TRADD* allele.

d, No GFP-expressing and hygromycin B/gancilcovir-resistant clones were found despite repeated attempts under varied experimental conditions.

n.a., not applicable.
